# Detection of Suspicious Cardiotocographic Recordings by Means of a Machine Learning Classifier

**DOI:** 10.3390/bioengineering10020252

**Published:** 2023-02-15

**Authors:** Carlo Ricciardi, Francesco Amato, Annarita Tedesco, Donatella Dragone, Carlo Cosentino, Alfonso Maria Ponsiglione, Maria Romano

**Affiliations:** 1 Department of Electrical Engineering and Information Technology (DIETI), University of Naples “Federico II”, 80125 Naples, Italy; 2 Department of Engineering for Innovation, University of Salento, 73100 Lecce, Italy; 3 Department of Experimental and Clinical Medicine ‘Gaetano Salvatore’, University “Magna Graecia” of Catanzaro, 88100 Catanzaro, Italy

**Keywords:** cardiotocography, support vector machine, fetal heart rate, machine learning

## Abstract

Cardiotocography (CTG) is one of the fundamental prenatal diagnostic methods for both antepartum and intrapartum fetal surveillance. Although it has allowed a significant reduction in intrapartum and neonatal mortality and morbidity, its diagnostic accuracy is, however, still far from being fully satisfactory. In particular, the identification of uncertain and suspicious CTG traces remains a challenging task for gynecologists. The introduction of computerized analysis systems has enabled more objective evaluations, possibly leading to more accurate diagnoses. In this work, the problem of classifying suspicious CTG recordings was addressed through a machine learning approach. A machine-based labeling was proposed, and a binary classification was carried out using a support vector machine (SVM) classifier to distinguish between suspicious and normal CTG traces. The best classification metrics showed accuracy, sensitivity, and specificity values of 92%, 92%, and 90%, respectively. The main results were compared both with results obtained by considering a more unbalanced dataset and with relevant literature studies in the field. The use of the SVM proved to be promising in the field of CTG classification. However, appropriate feature selection and dataset balancing are crucial to achieve satisfactory performance of the classifier.

## 1. Introduction

Cardiotocography (CTG) consists of the simultaneous recording of the fetal heart rate (FHR) and the uterine activity (uterine contractions, UC), respectively, using a Doppler probe and an indirect pressure transducer [1,2]. CTG can be considered an accurate diagnostic technique in evaluating the state of well-being of the fetus, as it is able to identify states of hypoxia (the decrease in oxygen to the fetus due to uterine contractions can further influence the FHR, which is directly related to the oxygen level, with possible harmful and irreversible consequences for the fetus itself) as early as possible, thus preventing further complications that can lead to fetal death or cerebral palsy. However, the evaluation of cardiotocographic traces is in fact highly subjective and qualitative if carried out through a visual inspection of the tracing, as still practiced in several countries [3,4].

To address this issue, the combination of computerized CTG along with the use of artificial intelligence (AI), with particular consideration for machine learning (ML), has been applied to several healthcare topics in medicine [5,6] and biomedical engineering [7,8,9] and has grown substantially also in the field of prenatal and perinatal diagnostics, especially for the early prediction of unhealthy conditions such as hypoxia, which can help clinicians in managing the pregnancy on time, thus avoiding unnecessary treatments, inappropriate actions or needless cesarean sections [10,11,12,13].

The most recent and well-known classifications of CTG traces use different definitions of the parameters representing the morphological characteristics of the FHR. The goal of defining “objective” and uniform parameters is mainly to obtain a significant predictive value for FHR monitoring and to enable evidence-based clinical management of intrapartum fetal compromise. On the other hand, however, each classification uses different definitions of the same parameters; hence, confusion and interpretation difficulties can be generated. While there is high/good agreement in the evaluation of clearly normal traces and obviously pathological traces, conversely there is a need to tackle the issue of suspicious traces, which represent a large portion of CTG. In these cases, it would be very useful to indicate the operation step by step, in order to be able to deal with the different clinical situations in the most appropriate way [14,15].

The classification methodologies proposed in the various studies carried out in the literature [16,17,18] differ from each other both in the application of techniques for the extraction of features necessary for a subsequent classification of CTG traces, and in the use and implementation of classification systems, using network architectures as close as possible to the problem under consideration. However, the works focused on quantitative and nonbiased algorithms for CTG evaluation are still limited [19] and, despite the importance of investigating suspicious CTG traces, the majority of the studies carry out binary CTG classification into normal and pathological signals, as the detection of pathological patterns necessitates for immediate delivery [20]. Therefore, the management of suspicious results remains unclear and less prescriptive, with wide variations across clinical centers [20]. Moreover, the precise interpretation of suspicious cases is fairly low by both visual and automated methods [21].

Based on what has been highlighted, in this work, a custom-made software application for the analysis and classification of CTG traces is described, a procedure for the automatic annotation of CTG traces is proposed, and a support vector machine (SVM) classifier of normal and suspicious CTG traces is implemented. The contribution of this work is three-fold: (i) it contributes to the investigation of suspicious CTG signals, which is still limited compared to the whole body of literature on CTG classification; (ii) it introduces a schematic approach for both the recognition of arrythmias in the FHR signal and the automatic annotation of suspicious CTG signals based on quantitative parameters extracted from the analysis of CTG traces; (iii) it shows promising performance metrics on the classification task, exceeding values present in the literature, even on a limited dataset.

## 2. Materials and Methods

In the following sections, the dataset and tools utilized for CTG classification are described in detail.

### 2.1. Data and Signals

CTG tracings, recorded by HP M11351A and SONICAID cardiotocographs during clinical routine monitoring, were retrospectively processed and elaborated by means of a custom-made software application for CTG analysis. Both signals underwent a pre-processing step as already described in [22,23,24] so that the same types of analyses could be performed regardless of the acquisition instrumentation. The original dataset used consisted of 580 CTG signals, mainly belong to healthy fetuses from the 24th to 42nd week of gestation. From a grand total of 580 CTG traces corresponding to fetuses, the dataset was filtered by including only the signals with the following characteristics: (i) CTG recorded in antepartum period between gestational weeks 36–40, thus excluding intrapartum CTG recordings; (ii) CTG duration > 20 min, as the minimum recommended duration in clinical practice; (iii) CTG with percentage of signal loss < 30% and outliers < 5%, in order to keep the cleanest signals and avoid those having bad quality; (iv) singleton pregnancies, thus excluding twin pregnancies, as they could be a source of confounding factors [25]. For the classification tasks, two sub-datasets from the original dataset were created, as explained in Section 2.5.

### 2.2. Software for the Analysis of CTG Traces

**Figure 1:**
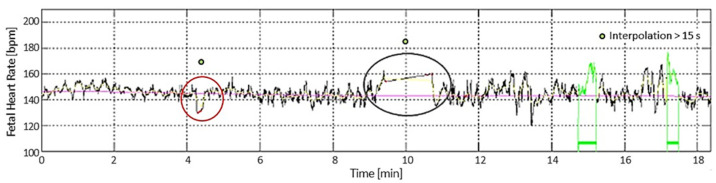
In the red circle, an example of interpolated segment (greater than 15 s), and in the grey oval, an example of a correctly excluded acceleration due to the long interpolation. FHR baseline is reported in magenta; the floating line, as defined in [26], is reported in yellow; accelerations are reported in green.

A custom-made software application dedicated to the automatic analysis of CTG traces has been developed. It allows one to analyze both “classic” parameters of the analysis carried out in the clinical setting and more innovative parameters. The software for the analysis of CTG signals allows the detection of a rather large amount of information relating to each trace, on the basis of which it is possible to obtain a more objective interpretation (Figure 1).

**Figure 2:**
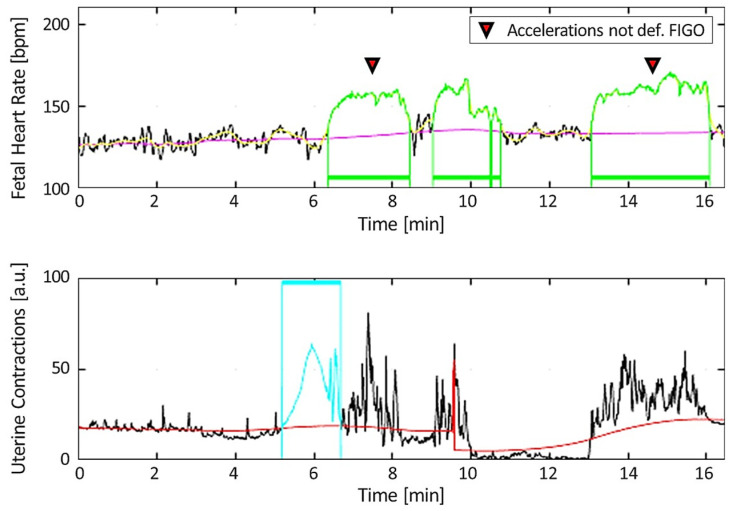
Graphical representation of an illustrative CTG trace with indication of acceleration falling outside the FIGO definition (indicated with triangles). FHR baseline is reported in magenta; the floating line, as defined in [26], is reported in yellow; accelerations are reported in green; contractions are reported in cyan; and UC basal tone is reported in red.

Furthermore, accelerations and decelerations not complying with FIGO guidelines [1,3,27] are highlighted with specific symbols (as shown in Figure 2).

### 2.3. Feature Extraction

Based on international guidelines, the previous literature and comparisons with a team of expert gynecologists [24,28], the features that were selected are as follows:

- Morphological characteristics: bradycardia, tachycardia, accelerations and decelerations.
- Characteristics in the Time Domain: Short-Term Variability (STV) index.
- Characteristics in the Frequency Domain: estimated value of the sympatho-vagal balance (SVB), expressed as the ratio between low-frequency and high-frequency power of the FHR variability (FHRV) signal, which respectively reflect mainly the activity of the sympathetic and vagal nervous systems.

The selected characteristics proved to be useful in determining the well-being of the fetus. Indeed, the presence of accelerations is generally considered an indicator of healthy fetuses, while the presence of decelerations, especially prolonged or variables ones, can indicate a pathological condition or suffering. The presence of prolonged and/or severe bradycardia or tachycardia can also represent an index of a pathological state. Furthermore, STV is closely related to a condition of fetal well-being; so, its decrease could lead to a severe impairment of the health of the fetus [29,30,31]. Finally, low SVB values reflect a condition of fetal non-reactivity, as the predominance of vagal activity indicates a condition of fetal non-reactivity compared to high SVB values that reflect fetal reactivity.

It is worth highlighting that, despite what we know regarding the importance of UC in the CTG analysis, in this work, we focused predominantly on the extraction of features related to the FHR signal.

#### 2.3.1. Morphological Features

**Figure 3:**
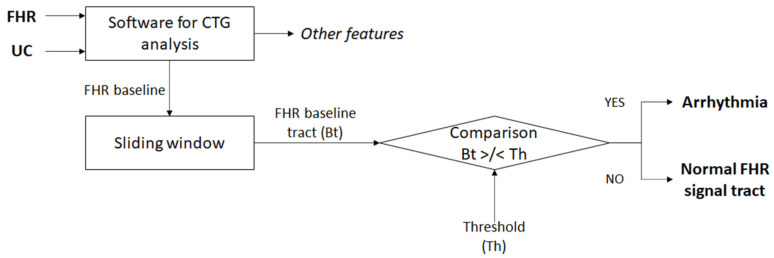
Simplified flow chart of the algorithm’s steps devoted to arrhythmia recognition. The threshold (Th) assumes different values depending on the type of arrhythmia to be detected (bradycardia or tachycardia). It is worth highlighting that this analysis was carried out on the FHR signal only; uterine activity (UC) was not processed for arrhythmia recognition.

In order to carry out a subsequent classification of the CTG traces on the basis of the characteristics described above, a function was implemented in Matlab to detect the sections of the FHR signal affected by possible cardiac arrhythmia. The calculation of bradycardia and tachycardia was performed on the baseline vector using a time interval reported in samples based on the values reported in the literature (Figure 3) [1,32].

Tachycardia is defined as a rise in the mean fetal heart rate above 160 bpm for at least 10 min; therefore, it was evaluated using an interval in samples corresponding to 10 min on the baseline vector, within which the samples were evaluated from time to time by comparing them with a threshold value, which in the case of tachycardia is 160 bpm. Subsequently, a condition was set according to which the function would return the vector of the stroke in which the tachycardia was detected, excluding zeros.

Bradycardia, on the other hand, is defined as a decline in mean fetal heart rate below 110 bpm for more than 3 min; therefore, it was evaluated using a sample time interval of 3 min. The procedure used was similar to that described for tachycardia: in this case, a threshold value of 110 bpm was used.

#### 2.3.2. Time Domain Feature Extraction

Once the morphological characteristics of the CTG traces had been extracted, the software computed some time domain features.

The parameter of interest to us was constituted, in particular, by the STV index.

**Figure 4:**
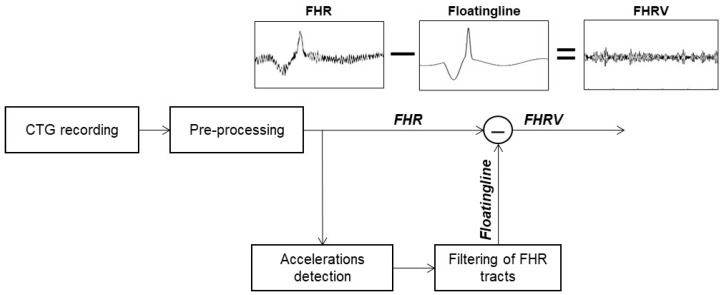
Schematic plot of the procedure developed for FHRV estimation. Details are reported in [15,26].

This index was evaluated using an algorithm step that, for each good-quality segment of the CTG, calculates the STV of the FHRV signal. It, in turn, was assessed by subtracting the floating line from the FHR signal; let us recall that the floating line is the median line of an FHR signal whose trend depends on the presence or absence of accelerations and/or decelerations [26,33]. Figure 4 depicts a scheme representing the procedure to compute floating line and FHRV signals.

Based on the extracted FHRV signal, the following equation was used to compute the STV [28,29]:

(1) [1n−1∑i=1n(F(i)−F¯)2]12

where $\bar{F}$ = FHR_mean_, *n* = number of beats (for real, uneven FHR series) or number of samples in 60 s (for evenly sampled FHR series; generally, output of CTG instrumentation).

**Figure 5:**
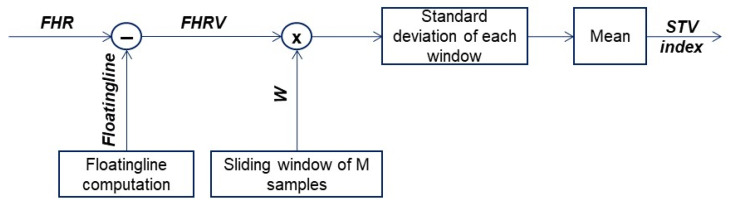
Flow chart of computation of the STV index.

The STV is calculated on 30 s segments of the FHRV signal; a 30 s analysis window is then used and translated over the entire signal, and the FHRV signal sections (corresponding to the 30 s windows) to be analyzed to estimate the STV are obtained by storing them in a cell array. The function returns the mean of the STV vector, obtained by calculating a value at each time window, following the flow chart shown in Figure 5.

#### 2.3.3. Frequency Domain Feature Extraction

The frequency domain analysis was carried out using the short time Fourier transform (STFT) to obtain a time-variant analysis of the power spectral density (PSD).

The following equation was used to compute the PSD [34]:

(2) $$P\left(\omega \right)=\frac{1}{LU}{\left|FT\left(\omega \right)\right|}^{2}$$

where *L* = window length; *U* = normalization factor; *FT* = Fourier transform.

In particular, the following equations were employed [35]:

(3) $$U=\frac{1}{L}\overset{L}{\underset{n=1}{\sum }}{\left[w\left(n\right)\right]}^{2}$$

(4) $$\bar{P\left(\omega \right)}=\frac{1}{nc}\overset{nc}{\underset{i=1}{\sum }}P_{i}\left(\omega \right)$$

(5) $$P=\frac{1}{N}\overset{N}{\underset{k=1}{\sum }}\bar{P\left(f_{k}\right)}$$

where $\bar{P\left(\omega \right)}$ is the mean PSD, and *k* is the number of samples on the frequency’s axis.

Of course, for each spectral band, the power was computed as an integral of the PSD. For this purpose, it was necessary to set a series of parameters:

- window type: Hanning;
- length of the window: 32 s;
- sampling step for interpolation: 0.25 s;
- number of points on which to calculate the spectrum: 1024;
- VLF band: 0–0.05 Hz;
- LF band: 0.05–0.2 Hz;
- HF band: 0.2–1 Hz.

**Figure 6:**
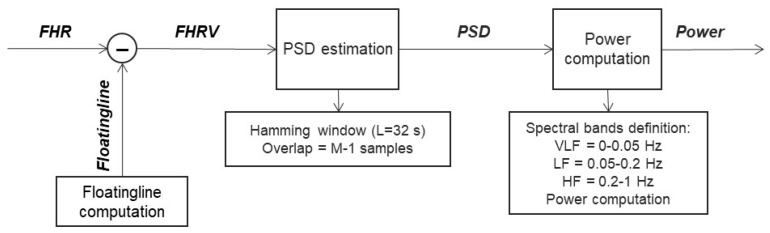
Flow chart of the computation of both PSD and power in the different spectral bands.

The flow chart followed for this algorithm’s steps is shown in Figure 6.

The analysis in the frequency domain using STFT allows one to obtain the following characteristics [36,37]:

- the estimated values of the powers in the different bands (VLF, LF and HF);
- the total power (given by the sum of the three values of VLF, LF and HF);
- the estimated value of the SVB (LF/HF); the higher the SVB, the more predominant the sympathetic activation compared to the vagal one.

An example of a signal’s features is reported in Table 1.

The characteristics used as input to the SVM classifier (bradycardia, tachycardia, number of accelerations and decelerations, STV and BSV) have shown good discriminating power in distinguishing the different CTG traces.

### 2.4. Machine-Based CTG Annotation

Having extracted the characteristics from the traces, the next step was to label the various signals analyzed on the basis of the values assumed by the parameters in normal conditions. For this purpose, threshold values were defined, and on that basis, a comparison was made for each parameter output from the analysis.

The threshold values of each characteristic were defined following the FIGO guidelines [1,3,27] for the values related to bradycardia and tachycardia and presence or absence of accelerations and decelerations. The parameters considered to be alarms can be summarized as follows:

- absence of accelerations
- presence of prolonged decelerations
- presence of severe tachycardia and/or bradycardia
- absence of variability (STV value < 0.02)

As far as the STV and SVB values, cut-off thresholds were evaluated by observing the trends in these indexes at different gestation weeks. In particular, the thresholds for STV and SVB corresponded to the minimum value assumed by the indicator, considered to be changing in the range between 24th and 42nd pregnancy week. The following cutoff values were thus obtained:

- STV threshold value: 1.70;
- BSV threshold value: 8.20.

**Figure 7:**
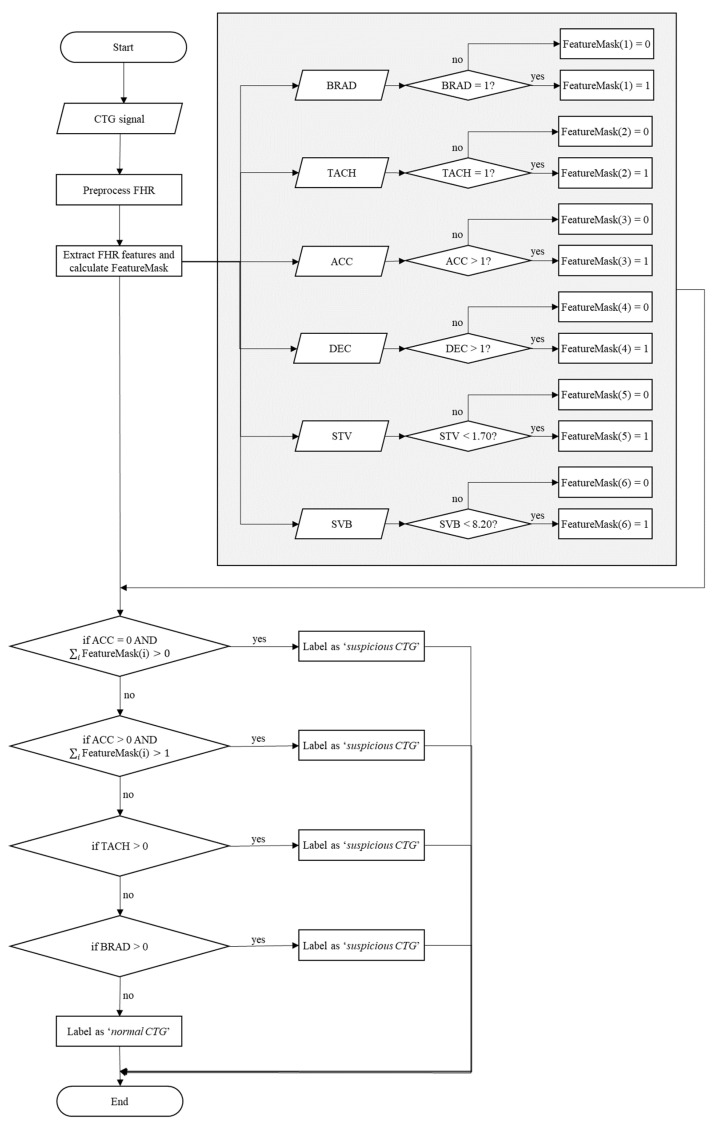
Flow chart of the machine-based CTG annotation algorithm.

On the basis of the conditions imposed, each signal considered was automatically annotated. Figure 7 illustrates a flow chart of the machine-based CTG annotation algorithm.

The characteristics saved in the previous analysis could thus be loaded by inserting the identification number of the corresponding trace. Then, a comparison was made between the input values or output parameters of the analysis and the threshold values. A feature mask was thus defined, containing the values of the characteristics 0 or 1 based on the conditions imposed. The criteria used for the identification of a suspicious trace were:

1. Absence of accelerations and at least a value equal to 1 in the feature mask;

OR

2. Presence of accelerations and at least two values equal to 1 in the feature mask;

OR

3. Presence of severe tachycardia (>180 bpm);

OR

4. Presence of bradycardia (<110 bpm).

In the case of suspicious characteristics, the labeling procedure was repeated for further verification. In particular, the traces were labeled on the basis of the values of the parameters extracted and differentiated according to two classes: normal traces and suspicious traces.

After the automated labeling of the traces, an SVM classifier was adopted to carry out the categorization of the CTG signals.

### 2.5. Support Vector Machine Classifier

The above-mentioned features were used as input to an SVM classifier. SVM, or kernel machines, are supervised learning methodologies for regression and pattern classification belonging to the family of generalized linear classifiers. They are also known as maximum margin classifiers, since at the same time they minimize the empirical classification error and maximize the geometric margin. The basic mechanism of the technique consists of identifying a hyperplane that separates the data belonging to two different classes. This hyperplane is determined by maximizing the geometric margin between the points of the two classes, or the optimal separation hyperplane that maximizes the distance between the hyperplane and the points closest to it of each class. The points located along the border are called support vectors [38]. The purpose of the application of the SVM is to use a predefined training set to build a model that decides to which class (among a finite set defined a priori) each sample of a test subset of data should be assigned based on the feature given as input to the classifier.

In this work, two classification tasks were carried out considering two subsets from the original dataset: (i) the first one consisting of 50 CTG traces, of which 30 were normal and 20 suspicious recordings (hereinafter referred to as Dataset 1); (ii) the second one still consisting of 50 CTG traces, of which 40 were normal and 10 suspicious (hereinafter referred to as Dataset 2). The two subsets of data were created in order to evaluate the accuracy of the classifier in detecting anomalous cases in both cases of balanced (Dataset 1) and unbalanced (Dataset 2) sets of data.

As far as the hyperparameters of the SVM, in this work a comparison between three different SVM kernels was carried out. Indeed, the kernel choice is a crucial step in the application of SVM classifiers, as it determines the extent and type of transformation of the space of the data. The classification performance of the following three different kernels, with increasing complexity from linear to radial based functions, were compared:

- Linear kernel
- Polynomial kernel (second order)
- Radial-based function (RBF) kernel

The results obtained with the tested kernels were compared to select the one achieving the best classification.

As far as the validation procedure, a 10-fold cross-validation [39] was chosen as the model evaluation method because, considering the set of data output from the analysis software, it was the one that allowed us to obtain the best results in terms of classification accuracy. The matrices of the samples were created to be used for training and testing. In the case considered, using a 10-fold cross-validation, we had 50 samples that we divided into 10 groups of five samples each, then we trained the classifier with nine groups (45 samples) and tested it with one group (five samples). This procedure was repeated 10 times: in this way, each group was used exactly once as a testing set. Finally, the 10 results (from the “folds”) were averaged in order to produce a single performance estimate.

The evaluation of the performance of the classifier was carried out by calculating the accuracy (and misclassification error) and the confusion matrix containing the values of true positives (TP), false negatives (FN), false positives (FP) and true negatives (TN) of the classification. From the confusion matrix, it was possible to derive two indices that separately evaluated the ability of the classifier to correctly classify positive and negative cases, i.e., the sensitivity (or recall), which is the ability to correctly classify the normal CTG traces: therefore, the fraction of the positive traces that were classified as positive, and the specificity, which is the ability to correctly classify the traces of the suspicious class, therefore, the fraction of the negative traces that were classified as negative. In addition, further metrics were calculated, namely the precision, which reflects the number of positive class predictions that actually belong to the positive class, the *F_β_ score*, which is used as a balance measure of both precision and sensitivity, and the geometric mean of the sensitivity and specificity (*G-mean*), as a balanced measure of both sensitivity and specificity. The above-mentioned performance metrics were calculated according to the following equations:

(6) $$Accuracy=\frac{TP+TN}{TP+FP+TN+FN}$$

(7) $$Sensitivity\;\left(Recall\right)=\frac{TP}{TP+FN}$$

(8) $$Specificity=\frac{TN}{TN+FP}$$

(9) $$Precision=\frac{TP}{TP+FP}$$

(10) $$F_{\beta }\;score=\left(1+\beta^{2}\right)\frac{Precision\cdot Sensitivity\;}{\left(\beta^{2}\cdot Precision\right)+Sensitivity\;}$$

(11) $$G\;mean=\sqrt{Sensitivity\;\cdot Specificity}$$

where *TP* = number of true positives, *TN* = number of true negatives, *FP* = number of false positives, *FN* = number of false negatives, and *β* is a positive real number (in this study, it was kept equal to 1 in order to assign the same importance to both precision and sensitivity metrics in the calculation of the *F_β_ score*).

Finally, the obtained classification results were compared with those obtained from some studies carried out in the literature.

## 3. Results

**Figure 8:**
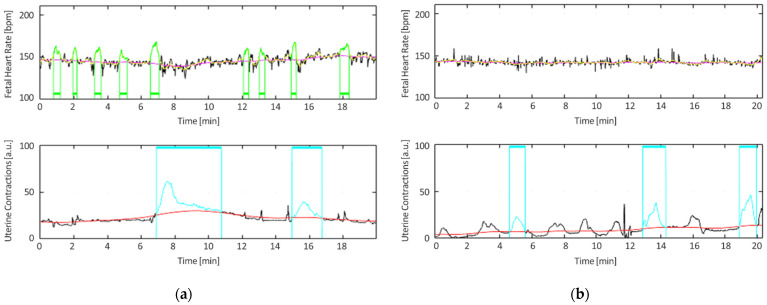
Examples of (**a**) normal and (**b**) suspicious CTG traces. FHR baseline is reported in magenta; the floating line, as defined in [26], is reported in yellow; accelerations are reported in green; contractions are reported in cyan; and UC basal tone is reported in red

Figure 8a,b and Table 2 provide a comparative illustration of a normal and suspicious trace.

As observed from the displayed figures, the suspicious trace reflected an overall absence of variability, which was confirmed by the values of the examined parameters for the classification, and consequently from the corresponding feature mask, as reported in Table 2.

The feature masks reported in Table 2 show how the normal CTG has a mask with all zero for each criterion, while the suspicious trace shows an absence of accelerations and at least a value equal to 1 in the feature mask.

**Figure 9:**
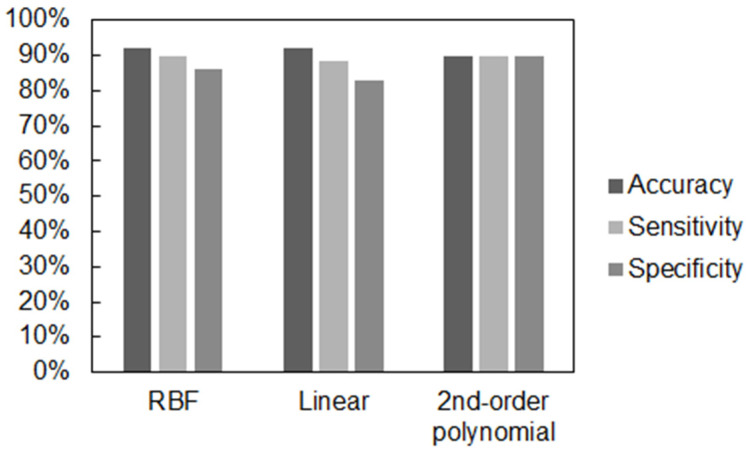
Average metrics obtained using different kernels.

The classification results on Dataset 1 (50 traces, of which 30 were normal and 20 suspicious) showed that the performance of the classifier was better using an RBF kernel, which turns out to be the most used and widespread in the case of classification using SVM. Figure 9 reports the values of the main performance metrics (accuracy, sensitivity, and specificity).

The classification results on Dataset 2, considering a more unbalanced dataset (40 normal and 10 suspicious traces) showed accuracy percentages similar to those obtained in Dataset 1 using an RBF kernel. However, as far as the sensitivity and specificity values, the results proved to be different probably due to the use of a more unbalanced set of data.

**Figure 10:**
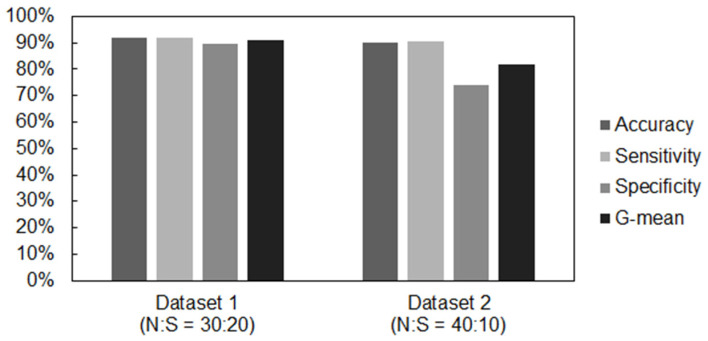
Overall performance results obtained with the two datasets examined.

The overall results of both studies carried out considering an RBF kernel are shown in Figure 10.

The values of sensitivity and specificity obtained in this case were around 90% and 74%, respectively. This means that, despite having obtained similar accuracy values, in Dataset 1, the classifier showed better performance. In fact, by calculating the value of the geometric mean (G-mean), obtained through the square root of the sensitivity value multiplied by that of specificity, in the first case a value of G-mean G = 90.8% was obtained, while in the second case we obtained a G value = 81.7%, thus confirming that better performance can be achieved with a more balanced dataset.

In Table 3 and Table 4, the confusion matrix and the values of the performance indicators for the best case are reported.

Overall metrics were calculated by averaging the values obtained for each class using both an arithmetic mean and a weighted mean (class size with respect to the total sample size was used as the weighting factor).

## 4. Discussion

The classification of CTG traces still remains a difficult task in clinical practice, as it is affected by strong intra and inter-observer variability. As already mentioned, the introduction of ML techniques in the field of CTG has as its main objective the early prediction of unhealthy fetal conditions in order to support clinical decision-making.

Various studies carried out in the literature show that some classifiers have promising performance in the CTG classification. For example, good results have also been obtained using those based on neural network classifiers, which showed high accuracy performance in comparison with classifications made by experts [40,41,42,43,44].

Liszka-Hackzell et al. [45] used a combined approach dividing the study into two parts: first, using unsupervised ML (SOM network) to map input vectors corresponding to CTG recordings (vectors with similar characteristics were mapped in the same region of the SOM network); second, a “back propagation” (BP) network was used to convert the X-Y coordinates obtained from the SOM network into the eight indices considered for CTG recording (bradycardia, tachycardia, absence of accelerations, reduced variability, presence of distinct decelerations in variables, early and late, a normal pattern). The correct diagnosis percentages obtained for each index showed values of 100% for bradycardia and tachycardia, 97% for the normal pattern, and values higher than 80% for the other indices, while the lowest value was obtained for variable decelerations, of just over 60%.

Cazares et al. [46] proposed an approach for the classification of CTG recordings using as characteristics the vectors describing the morphology of the accelerations and decelerations (mean values of the FHR baseline and the amplitudes, areas, durations and delay times), which are mapped in a two-dimensional viewing space. A pre-clustering (K-means) algorithm was proposed in order to reduce the number of vectors to be supplied as input to an RBF network, normalizing each vector characteristic. The vectors mapped in the two-dimensional display space were grouped as follows: the “normal” vectors mainly in the upper left quadrant of the space, and the “abnormal” ones mostly in the lower right quadrant, with a spread of “suspicious” vectors between the two regions. These results demonstrated a correspondence in comparison with evaluations by an expert, thus providing a decision support tool in the evaluation of CTG patterns in the absence of an expert.

Georgoulas et al. [40] proposed a classifier based on feed-forward neural networks (MLP) to categorize the CTG traces, introducing a step to reduce the size of the input data using independent component analysis (ICA) as a preprocessing stage in order to simplify and reduce the complexity of the problem under consideration. Twenty signals were classified (19 for training and 1 for testing), with the procedure repeated 20 times and the results averaged. The performance of the classifier was shown to be better using a minimum number of features, obtaining an accuracy value of 85%.

The proposed classification methodology using a SVM classifier has shown good performance in categorizing CTG tracings. The traces selected from a large database of real signals recorded and acquired in the antepartum periods were labeled according to two classes: normal and suspicious. The traces belonged almost entirely to normal fetuses, among which some suspicious cases were identified with low values of the parameters considered in relation to the normal conditions imposed. A binary classification of the tracings was thus proposed, obtaining promising results both as regards the accuracy value of the classifier and the sensitivity and specificity indices; in particular, considering a set of traces (Dataset 1) consisting of 30 normal and 20 suspicious signals, the classifier showed better performance, with accuracy, sensitivity and specificity values around 92%, 92%, and 90%, respectively. In the case of a more unbalanced dataset (Dataset 2), i.e., considering 40 normal and only 10 suspicious traces, while good overall performance (accuracy equal to 90%) was achieved, significant differences were observed in the values for sensitivity (90%) and specificity (74%) compared to the previous case. The results are shown in Table 5.

The characteristics used as inputs to the SVM classifier (bradycardia, tachycardia, number of accelerations and decelerations, STV and BSV) have shown good discriminating power in distinguishing different CTG traces.

**Figure 11:**
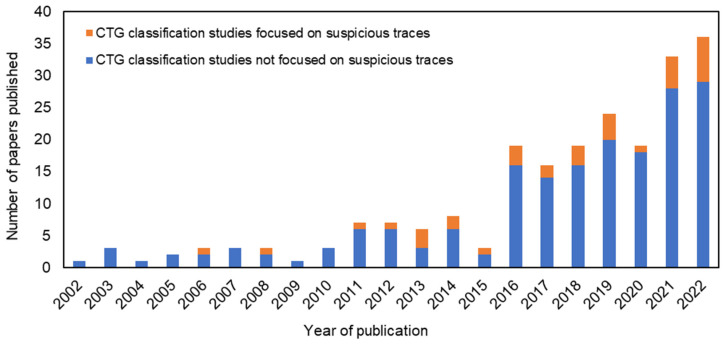
Number of papers on CTG classification published in the last 20 years with indication of those focused on suspicious CTG recordings. The literature search was carried out through Elsevier Scopus database with the following keywords in both title and abstract: (“cardiotocography” OR “CTG”) AND (“classification” OR “machine learning”) AND (“suspicious” OR “suspect” OR “risky”) to search for papers focused on suspicious CTG recordings; (“cardiotocography” OR “CTG”) AND (“classification” OR “machine learning”) to search for all papers on CTG classification.

The different approaches proposed in the literature do not allow a direct comparison due to the different sets of characteristics used and different methods of extracting the characteristics. In addition, so far, binary classifications have been proposed mainly considering two states of the fetus: “normal” and “abnormal”. Indeed, from a literature search carried out through Elsevier Scopus database, it appeared that even though the number of papers published on CTG classification has significantly increased in the last 20 years, the works focused on suspicious CTG signal classification represent a very small percentage (average < 15%) of all manuscripts published, as shown in Figure 11.

From a comparative analysis, we observed that our approach yielded similar or even better performance than those reported by studies in the literature using SVM algorithms for the binary classification of CTG traces. Krupa et al. [47] adopted SVM for 90 traces divided into two independent sets, a training set of 60 traces, of which 40 were “at risk” and 20 “normal”, and a testing set of 30 traces, of which 20 were “at risk” and 10 “normal”. Normal signals were labeled as “positive”, while “at risk” were labeled as “negative”. Magenes et al. [48] instead obtained better accuracy than using a second-order polynomial kernel, using the classifier on a dataset of 100 traces, of which 70 were used for the training set and 30 for the testing set. In this case, the tracings belonging to suffering fetuses were labeled as “positive”, and those belonging to normal fetuses were labeled as “negative”. Finally, Georgoulas et al. [39] labeled the traces on the basis of the pH value of the fetal umbilical artery blood, considering “normal” those with a pH value < 7.10 and “at risk” those with a pH value > 7.20. Additionally, in this case the traces belonging to “at risk” fetuses were considered “positive” and the normal ones as “negative”. The dataset used consisted of 80 CTG recordings, of which 60 were normal and 20 were at risk; the signals were divided into 10 subsets containing eight cases each (six normal and two at risk). The characteristics used were organized into several subsets, which included two different sets for the morphological characteristics (Mset1, Mset2), a set of characteristics comprising seven parameters in the time domain (Tset) and two different sets for the characteristics in the frequency domain (Fset1, Fset2). Principal component analysis (PCA) was used to reduce the size of the features used, evaluating the set of features that demonstrated the best performance. The results observed on 20 min FHR signal segments showed better performance using the “Tset, Fset2” set.

The results of the different approaches proposed in the literature described above are summarized in the following Table 6.

It is worth highlighting that the different approaches proposed in the literature do not allow a straightforward comparison with the model proposed in this work. Despite this, from the results obtained, it can be observed that the performance of SVM classifiers is promising and, therefore, their future clinical application in the field of CTG is envisaged since it could bring advantages in overcoming the strong inter-observer and intra-observer variability linked to the visual inspection of CTG traces.

Although the results obtained showed that the SVM classifier could be a good future candidate in the classification of CTG traces, there were some aspects that should be taken into consideration both as limitations of the present study and as future developments in the research:

- first of all, the classifier was not trained to recognize particular patterns, which included, for example, traces characterized by increased variability (>25 bpm) or sinusoidal patterns, which are typical characteristics of pathological traces;
- in the present study, only the presence or absence of decelerations was evaluated; however, as future directions, different morphologies and durations of the decelerations also can be considered to accurately classify the different types of tracings;
- the value of the Apgar index at birth could be considered as an additional aspect in the future;
- the behavioral state of the fetus should be considered, since often the absence of accelerations is linked to a state of rest of the fetus;
- on the basis of the normal conditions imposed by an evaluation of the STV and SVB indices, reference should also be made to the gestation week in further studies;
- the potential of other, more effective machine learning algorithms for both regression (e.g., SVR [49,50,51]) and classification tasks could be exploited in the future;
- finally, the dataset considered in this work included only two groups of signals, but in future research works, the classification will be extended by including CTGs with different characteristics and taking advantage of open access databanks with both annotated and nonannotated signals from healthy, suspect or pathologic subjects.

## 5. Conclusions

Although the literature on CTG is very broad, even the introduction of computerized CTG has not been able to bring concrete improvements or objective and conclusive results in terms of CTG analysis and classification. Moreover, it is worth highlighting that, in some countries, CTG is a diagnostic exam with legal value. Therefore, research in this field is even more important. Furthermore, there are still very few studies focusing on the accurate recognition of suspicious tracings, which has led to additional diagnostic tests with a consequent increase in health expenditures. Thus, the accurate recognition of suspicious CTGs an important from both the clinical and socio-economic perspectives.

The present work provided a contribution in this direction, as it contributes to the study and classification of suspicious CTG signals, whose investigation represents a very limited percentage (<15%) of the total number of published works on CTG classification. In addition, we proposed a schematic approach both for the recognition of arrythmias in the FHR signal and for the automatic annotation of suspicious CTG signals based on quantitative parameters extracted from the analysis of the CTG trace. Finally, the proposed application of an SVM classifier showed promising results in the binary classification of CTG traces, with performance metrics (up to 92% accuracy) exceeding those reported in the reference literature. A possible future use of this type of classifier could bring advantages in the field of CTG as a support tool for the interpretation and classification of the recordings.

In the future, the prospective use of a classifier in CTG clinical practice could constitute a valuable decision support system for gynecologists, since it can lead to a faster and less variable classification of CTG traces on the basis of more objective diagnostic parameters, closely related to fetal well-being, that cannot be detected by a simple visual inspection of the signals.

## Figures and Tables

**Table 1:** Characteristics extracted from a representative CTG trace from the dataset (BL = baseline; BRAD = bradycardia; TACH = tachycardia; ACC = accelerations; DEC = decelerations; UC = uterine contractions; STV = short term variability; VLF, LF, HF = power in very low, low, and high frequency band; SVB = sympatho-vagal balance).

BL	BRAD	TACH	ACC	DEC	UC	STV	VLF	LF	HF	SVB
136.63	0	0	8	0	2	1.46	4.36	2.61	0.22	11.6

**Table 2:** Example of features’ values and features’ masks obtained for a normal and suspicious CTG trace (BRAD = bradycardia; TACH = tachycardia; ACC = accelerations; DEC = decelerations; STV = short term variability; SVB = sympatho-vagal balance).

Type of CTG		BRAD	TACH	ACC	DEC	STV	SVB
Example of a normal CTG trace	Parameter values	0	0	9	0	2.77	8.88
	Corresponding feature mask	0	0	0	0	0	0
Example of a suspicious CTG trace	Parameter values	0	0	0	0	1.72	1.71
	Corresponding feature mask	0	0	1	0	0	1

**Table 3:** Confusion matrix for the best case considered.

	Predicted Normal	Predicted Suspicious
Actual normal	29	1
Actual suspicious	3	17

**Table 4:** Performance metrics for the best case considered.

	Accuracy (%)	Misclassification Error (%)	Sensitivity (%)	Specificity (%)	Precision (%)	F1 Score
Normal class	-	-	96.7	85.0	90.6	0.935
Suspicious class	-	-	85.0	96.7	94.4	0.894
Overall (arithmetic mean)	92.0	8.0	90.8	90.8	92.5	0.914
Overall (weighted mean)	92.0	8.0	92.0	89.7	92.1	0.919

**Table 5:** Main results obtained from the classification using the SVM in the present study.

	Accuracy	Sensitivity	Specificity
Dataset 1	92.0%	92.0%	89.7%
Dataset 2	90.0%	90.4%	73.9%

**Table 6:** Results of similar studies carried out in the literature using the SVM to classify CTG traces.

	Accuracy	Sensitivity	Specificity
Krupa et al. [47]	86.0%	94.8%	70.0%
Magenes et al. [48]	78.0%	78.0%	79.0%
Georgoulas et al. [39]	81.2%	70.0%	85.0%

## Data Availability

The data presented in this study are available on request from the corresponding author.
